# Fungicides and insecticides can alter the microbial community on the cuticle of honey bees

**DOI:** 10.3389/fmicb.2023.1271498

**Published:** 2023-10-30

**Authors:** Fabienne Reiß, Antonia Schuhmann, Leon Sohl, Markus Thamm, Ricarda Scheiner, Matthias Noll

**Affiliations:** ^1^Institute of Bioanalysis, Coburg University of Applied Sciences and Arts, Coburg, Germany; ^2^Behavioral Physiology and Sociobiology, Biocenter, Julius Maximilian University of Würzburg, Würzburg, Germany; ^3^Bayreuth Center of Ecology and Environmental Research (BayCEER), University of Bayreuth, Bayreuth, Germany

**Keywords:** pesticides, bee, cuticular microbiome, fungi, bacteria, plant protection products, neonicotinoids

## Abstract

Honey bees are crucial for our ecosystems as pollinators, but the intensive use of plant protection products (PPPs) in agriculture poses a risk for them. PPPs do not only affect target organisms but also affect non-targets, such as the honey bee *Apis mellifera* and their microbiome. This study is the first of its kind, aiming to characterize the effect of PPPs on the microbiome of the cuticle of honey bees. We chose PPPs, which have frequently been detected in bee bread, and studied their effects on the cuticular microbial community and function of the bees. The effects of the fungicide Difcor^®^ (difenoconazole), the insecticide Steward^®^ (indoxacarb), the combination of both (mix A) and the fungicide Cantus^®^ Gold (boscalid and dimoxystrobin), the insecticide Mospilan^®^ (acetamiprid), and the combination of both (mix B) were tested. Bacterial 16S rRNA gene and fungal transcribed spacer region gene-based amplicon sequencing and quantification of gene copy numbers were carried out after nucleic acid extraction from the cuticle of honey bees. The treatment with Steward^®^ significantly affected fungal community composition and function. The fungal gene copy numbers were lower on the cuticle of bees treated with Difcor^®^, Steward^®^, and PPP mix A in comparison with the controls. However, bacterial and fungal gene copy numbers were increased in bees treated with Cantus^®^ Gold, Mospilan^®^, or PPP mix B compared to the controls. The bacterial cuticular community composition of bees treated with Cantus^®^ Gold, Mospilan^®^, and PPP mix B differed significantly from the control. In addition, Mospilan^®^ on its own significantly changed the bacterial functional community composition. Cantus^®^ Gold significantly affected fungal gene copy numbers, community, and functional composition. Our results demonstrate that PPPs show adverse effects on the cuticular microbiome of honey bees and suggest that PPP mixtures can cause stronger effects on the cuticular community than a PPP alone. The cuticular community composition was more diverse after the PPP mix treatments. This may have far-reaching consequences for the health of honey bees.

## 1. Introduction

A significant decline in pollinators has been observed in the past decade, despite animal pollination being one of the most important ecosystem services (Potts et al., 2010). One of the main drivers that could lead to this pollinator decline is the intensive use of plant protection products (PPPs) in agriculture (Kluser and Peduzzi, 2007; Goulson et al., 2015). For the investigation of adverse side effects of PPPs on beneficial insects, the honey bee *Apis mellifera* is an excellent model organism because of its rich behavioral repertoire and the large diversity of methods for investigating their behavior (Scheiner et al., 2013).

Among the different PPP groups, insecticides are the best-studied class as they often show negative effects on beneficial insects (Galvan et al., 2006; El Hassani et al., 2008; Shi et al., 2019). Fungicides are usually not sufficiently investigated since no harmful impact on insects is assumed (Zubrod et al., 2019; Schuhmann et al., 2022). Nevertheless, some fungicides can synergistically interact with insecticides or prolong their undesirable effects on the health of pollinators (Wernecke and Castle, 2020). This study tested combinations of PPPs frequently detected in bee bread (Rosenkranz et al., 2019). The insecticide Steward® was combined with the fungicide Difcor®, and the insecticide Mospilan® was applied together with the fungicide Cantus® Gold (Table 1). Both combinations have been used in agriculture and are consumed by bees as they are applied to mass-flowering crops (Holzschuh et al., 2013).

**Table 1:** Overview of the plant protection products (PPPs) used (FMC Agricultural Solutions, 2020, 2021; BASF SE, 2021; Fungicide Resistance Action Committee, 2021; Insecticide Resistance Action Committee, 2021; PLANTAN GmbH, 2021).

	PPP	Authorization holder	Active ingredient	Group	Area of application
Mix A	Difcor®	Globachem NV, Sint-Truiden, Belgium	Difenoconazole	SBI* fungicide	Fruit growing
	Steward®	Cheminova Deutschland GmbH & Co. KG, Stade, Germany	Indoxacarb	Oxadiazine	Fruit growing
Mix B	Cantus® Gold	BASF SE, Ludwigshafen, Germany	Boscalid + Dimoxystrobin	Succinate-dehydrogenase inhibitor + quinone outside inhibitors	Rapeseed cultivation
	Mospilan®	Nisso Chemical Europe GmbH, Düsseldorf, Germany	Acetamiprid	Neonicotinoid	Rapeseed cultivation

*Sterol biosynthesis inhibitors.

Indoxacarb is the active ingredient of the insecticide Steward® and acts as a sodium channel modulator, leading to a quick inhibition of feeding in pest insects (Börner et al., 2009). Acetamiprid (active ingredient of the insecticide Mospilan®) is a neonicotinoid that acts as an agonist on nicotinic acetylcholine receptors, resulting in constant ion flow and neurotoxic effects (Börner et al., 2009). Several negative side effects of the isolated application of both insecticides on beneficial insects have already been demonstrated (Galvan et al., 2006; Laurino et al., 2011; Shi et al., 2020). In contrast to these insecticides, the fungicides difenoconazole (an active ingredient of Difcor®) and boscalid or dimoxystrobin (active ingredients of Cantus® Gold) appear to have small or no effects on beneficial insects when applied on their own (Wernecke et al., 2019; Almasri et al., 2020).

Bees are often exposed to a mixture of several PPPs. This can result from tank mixtures, spraying sequences, the combined use of seed and spray treatment, or bees foraging sequentially at different flowers (Thompson et al., 2014). Thus, various active substances are present in bee bread (Rosenkranz et al., 2019). It has been shown that certain insecticide–fungicide combinations can lead to synergistic negative effects (Schuhmann et al., 2022), which makes it important to study more intensively the interaction of insecticides and fungicides in insects.

PPPs affect not only pest organisms but also non-targets such as bees and microorganisms in the environment. Honey bees come in contact with a wide variety of microbiomes, namely pollen (Gilliam et al., 1989; Martinson et al., 2011), nectar (Fridman et al., 2012; Alvarez-Pérez and Herrera, 2013), bee bread (Sinpoo et al., 2017), and plants’ surfaces (Keller et al., 2021). Nevertheless, little is known about the cuticular microbiome of bees and its effects on the health and performance of honey bees. In contrast, the gut microbiome is well studied since it plays a central role in metabolism, growth, development, protection against pathogens, and immune defense (Zheng et al., 2017; Kwong et al., 2017a).

The core gut microbiome of honey bees is dominated by nine bacterial species clusters, which account for 95 to 99.9% of the bacteria in almost all individuals (Babendreier et al., 2007; Engel et al., 2012; Moran et al., 2012; Sabree et al., 2012; Corby-Harris et al., 2014). Five core bacterial species clusters and four rarer species clusters form the dominant cluster of bacteria found in honey bees (Kwong et al., 2017b; Raymann and Moran, 2018). The four core bacteria are *Snodgrassella alvi*, *Gilliamella apicola*, *Lactobacillus* Firm-4*, Lactobacillus* Firm-5, and *Bifidobacterium asteroids* (Babendreier et al., 2007; Martinson et al., 2011; Kwong and Moran, 2013).

The gut mycobiome of most worker bees is dominated by the members of the genus *Saccharomyces*, whereas the intestines of forager bees and queens were colonized by various fungal taxa, including *Zygosaccharomyces*, *Candida,* and *Ascomycota*. Katsnelson (2015) pointed out that the gut microbiome is not the only microbial community that is important for bees. For example, bees have close contact with bacteria on the inner walls of the hive (Anderson et al., 2013).

The gut of the honey bee and whole-body extracts appeared to have the same dominant microbiomes but vary in relative abundance and composition (Mattila et al., 2012; Ribière et al., 2019). Aizenberg-Gershtein et al. (2013) analyzed the cuticular bacterial community composition. The cuticular bacterial microbiome is colonized by the bacterial classes *Gammaproteobacteria*, *Actinobacteria*, *Bacilli,* and *Alphaproteobacteria*. *Arsenophonus* represented the most dominant genus within *Gammaproteobacteria*. *Arsenophonus* represents an extensive cluster of symbiotic bacteria in insects (Nováková et al., 2009), which has already been recovered in the gut of honey bees (Babendreier et al., 2007). Saccà and Lodesani (2020) identified and isolated bacteria (*Apilactobacillus kunkeei*, *Bacillus thuringiensis*, and *Acetobacteraceae*) from the cuticle of honey bees, which might function as a natural antagonist of the external parasitic mite *Varroa destructor*.

The study herein aimed to analyze (i) the effects of single neonicotinoids and fungicides; (ii) the effects of a mixture of a non-neonicotinoid (Steward®) with an SBI fungicide (Difcor®) (mix A, Table 1), and (iii) the effects of a combination of a neonicotinoid (Mospilan®) and a non-SBI fungicide (Cantus® Gold) (mix B, Table 1) on the bacterial and fungal communities of honey bees. The quantity, composition, and function of the different microbes were investigated. We hypothesize that fungal taxa and, to a lesser extent, bacterial taxa will be reduced in abundance, and some taxa might even be sensitive to PPPs. On the other hand, we expect that other microbes will gain an advantage due to the inactivation of competitors and the addition of PPPs as substrates.

## 2. Methods

### 2.1. Treatment of honey bees

**Figure 1:**
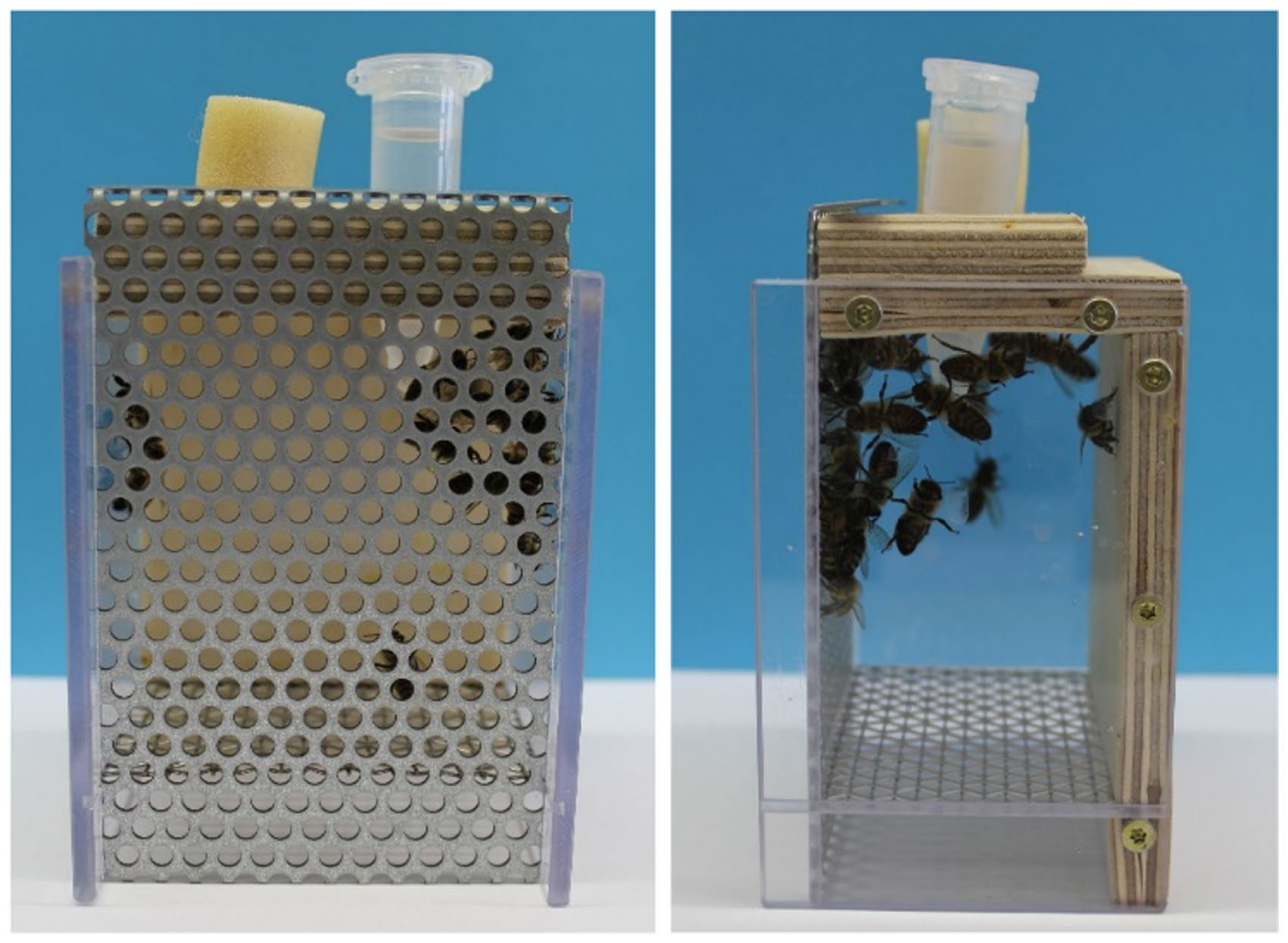
Experimental setup of the bee’s treatment. Experimental setup of *Apis mellifera* nursing bees in a cage system. The feeding solution is provided via a prepared 2 mL cup with holes at the bottom. Picture A. Schuhmann.

Honey bee workers (*A. mellifera carnica*) were collected randomly from a hive in the departmental apiary at the University of Würzburg. For each treatment, five cages were prepared on five consecutive days. Each cage contained 30 honey bees. The cages were maintained in an incubator (30°C, 50% humidity), and the honey bees received the treatment solutions for 1 week. The feeding solutions were provided via prepared 2 mL cups (Figure 1). The amount of food per cage was adapted to the number of individuals so that the bees could eat *ad libitum*. The 2 mL cups were replaced every day to guarantee a controlled food supply.

The feeding solutions were calculated based on residue levels found in the field and consisted of 30% sugar water with the addition of PPPs (McArt et al., 2017; Lüken and von der Ohe, 2018; El-Nahhal, 2020; Friedle et al., 2021). For reasons of data availability, we relied on residue values from pollen for the calculation of Steward® and Difcor® and on residue values from honey for Mospilan® and Cantus® Gold.

Using the intake of honey or pollen per bee per day (Rortais et al., 2005), it was calculated how much active ingredient a honey bee would consume per day based on the selected residues. It was assumed that a caged honey bee consumes 60 μL (Hesselbach and Scheiner, 2019) of feeding solution per day. Therefore, the feeding concentration was adjusted so that a honey bee would ingest the corresponding calculated active ingredient concentration in these 60 μL (Table 2).

**Table 2:** Overview of plant protection product (PPP) treatments.

PPP treatments	Residue [μg/kg]	PPP concentration in the feeding solution [μg/L]	Average of PPP consumed per day per bee in a cage [μg]
Difcor®	48 (in pollen) (46)	9	0.000576
Steward®	557 (in pollen) (43)	100	0.006
Mix A (Difcor® + Steward®)	48 (in pollen) (46) 557 (in pollen) (43)	9 + 100	0.000576 + 0.006
Control A		0	0
Cantus® Gold	5 (in honey) (44)	10	0.0008
Mospilan®	72.5 (in honey) (45)	200	0.012
Mix B (Cantus® Gold+ Mospilan®)	5 (in honey) (44) 72.5 (in honey) (45)	10 + 200	0.0008 + 0.012
Control B		0	0

Doses and their corresponding residue values in the field based on which the concentrations were calculated are indicated. Feeding solution consists of 30% sugar water and the addition of PPP.

We used the water-soluble formulation of PPPs to mimic field conditions (Cox and Surgan, 2006). The honey bees were either treated with the insecticide alone, the fungicide alone, a mixture of both, or with a control solution (30% sugar water). All four treatments belonged to one experimental series (Table 2). Each treatment group had its own control (“control A” belongs to Difcor®, Steward®, and PPP mix A treatments; “control B” belongs to Cantus® Gold, Mospilan®, and PPP mix B treatments). The concentrations of the single PPPs were maintained in the mix A and B treatments. After the 1-week treatment, the bees were frozen in liquid nitrogen and stored at −80°C.

### 2.2. Cuticle preparation and DNA extraction

Dissection and preparation of cuticles were performed under frozen conditions. Antennae, legs, and wings were removed (but not discarded). Afterward, the inner organs (i.e., brain, muscles, gut, and sting apparatus) were carefully removed from the head capsule, the thorax, and the abdomen (Ribière et al., 2019; Subotic et al., 2019). The prepared cuticles and outer body parts underwent DNA isolation. Cuticles from four individuals were pooled for one sample. DNA extraction was performed using the Quick-DNA™ Fecal/Soil Microbe Microprep Kit according to the manufacturer’s protocol (Zymo Research Europe GmbH, Freiburg im Breisgau, Germany). All DNA extracts were stored at −20°C until further use. Five independent bee replicates per treatment were analyzed.

### 2.3. Quantitative PCR of bees cuticular DNA extracts

The quantitative PCR (qPCR) was performed to quantify the gene copy numbers of the bacterial 16S rRNA gene with the primer sets BAC341f (5′-CCTACGGGNGGCWGCAG-3′) and BAC758R (5′-GACTACHVGGGTATCTAAKCC-3′) (Klindworth et al., 2013) and of the fungal internal transcribed spacer (ITS) DNA regions with the primer sets fITS7 (5′-GTGAATCATCGAATCTTTG-3′) (Ihrmark et al., 2012) and ITS4 (5′-TCCTCCGCTTATTGATATGC-3′) (White et al., 1994). Each independent replicate was quantified in three technical triplicates in 96-well plates using the CFX96™ Real-Time System C1000™ Thermal Cycler (Bio-Rad Laboratories GmbH, Feldkirchen, Germany). Fungal ITS and bacterial 16S rRNA gene-base qPCR were performed in 20 μL reaction mixtures containing 1 μL of DNA template, 0.3 μM each primer, 1x PCR-Enhancer (Biozym Scientific GmbH, Oldendorf, Germany), 1x iTaq Universal SYBR Green Supermix (Bio-Rad, Munich, Germany), and nuclease-free water (Sigma-Aldrich, St. Louis, MO, USA). Nuclease-free master mix blanks were run as negative controls. Reaction conditions for 16S qPCR involved an initial 3-min denaturation at 95°C, followed by 40 cycles of 5 s of denaturation at 95°C, annealing at 52°C for bacteria and 52.7°C for fungi over 30 s, respectively, and elongation at 60°C for 30 s. The final elongation step was at 72°C for 10 min. Gene copy numbers were calculated as previously described by Lasota et al. (2019) by comparing PCR-cycle threshold (CT) values to a standard curve of triplicate 10-fold dilutions of genomic DNA (gDNA) extracted from a known concentration of *Escherichia coli* K12 (DSM 423) and *Fusarium solani* (DSM 1164) by employing the Quick-DNA™ Fecal/Soil Microbe Miniprep Kit according to the manufacturer’s instructions (Zymo Research Europe GmbH). The genomic DNA concentration per PCR reaction of *E. coli* and *F. solani* standard ranged from 1 × 10^9^ to 5 × 10^3^ and 6.51 × 10^6^ to 65.1 gene copies.

### 2.4. Amplicon sequencing of the cuticular microbiome

Cuticular DNA samples were further analyzed by amplicon sequencing. The 16S rRNA gene and ITS DNA region were amplified with the same primer sets used for the qPCR analysis to create amplicon sequencing libraries for each of the 40 bees’ cuticular DNA samples. Inline barcodes and Illumina sequencing adapters were added to the amplicon sequence libraries using the Nextera CT Index Kit (Illumina, San Diego, CA, USA) and MiSeq Reagent Kit v3 600 cycles (Illumina) according to the manufacturer’s instructions. PCR products for library preparation were purified by AMPure XP beads (Beckman Coulter, Brea, CA, USA). The sequencing of libraries was performed by 300-bp paired-end sequencing on an Illumina MiSeq platform (Illumina MiSeq V3; Illumina) based on a standard protocol from the manufacturer. Amplicon sequencing library preparation, sequencing, and sequence quality checks were carried out by LGC Genomics GmbH (Berlin, Germany).

### 2.5. Bioinformatics

Raw data pre-processing with demultiplexing, sorting, adapter trimming, and merging reads was congregated using Illumina bcl2fastq conversion software v2.20 and BBMerge v34.48 (DOE Joint Genome Institute, 2022). The sequence quality of the reads was controlled with the FastQC software, version 0.11.8 (Babraham Bioinformatics, 2020). Sequence pre-processing and Operational Taxonomic Units (OTUs) picking from amplicons were conducted using Mothur 1.35.1 (Schloss et al., 2009). Sequences were aligned against the 16S Mothur-Silva SEED r119 reference alignment depending on their Phred quality score over 33 (Whelan et al., 2019). Filtering short alignments and reducing sequencing errors were conducted by pre-clustering, where a maximum of one base mismatch per 100 bases within a cluster was allowed. Chimeras were eliminated with the UCHIME algorithm (Edgar et al., 2011). Afterward, taxonomical classification of the sequences against the Silva reference classification was conducted, and sequences of other domains of life were removed for OTU picking. OTUs were selected and assigned to a taxonomic level by clustering at the 97% identity level (Edgar, 2018; Nilsson et al., 2019; Kõljalg et al., 2020). Thereby, OTU tables for DNA samples were constituted.

Ecological and metabolic functions of detected bacterial OTUs were predicted using the functional annotation tool of the prokaryotic taxa v.1.1 (FAPROTAX) database (Louca et al., 2016). The functions of each prokaryotic taxon were annotated using the literature on cultivable strains. The FungalTraits database (Põlme et al., 2021), a specific functional prediction tool, was used to taxonomically parse fungal genera by ecological guild independent of the sequencing method. Bacterial and fungal function count tables for each DNA sample were generated. Sequence counts of OTUs for fungi ranged from 919 to 345,516 and from 3,131 to 204,067 for bacteria.

### 2.6. Statistics

Statistical analyses were performed after OTUs were taxonomically summarized at the genus level. The normal distribution of each dataset was tested via OriginPro 2022 (OriginLab Corporation, Northampton, MA, USA) by the Shapiro–Wilk test (*p* < 0.05). Rarefaction analysis as well as the estimation of alpha diversity (OTU richness, Shannon index, Simpson index, and Pielou’s Evenness) and OTU richness estimators [bias-corrected Chao1 and an abundance-based coverage estimator (ACE)] were performed for cuticular DNA samples in RStudio (Version 2022.02.1, RStudio, Inc., Boston, MA, USA) and the package vegan 2.5–7 (Oksanen et al., 2022; R: The R Project for Statistical Computing, 2022).

Alpha diversity indices were tested for normal distribution by Shapiro–Wilk test (*p* < 0.05). Significant effects (*p* < 0.05) on cuticular alpha diversity indices for each PPP treatment were calculated either by one-way ANOVA with a post-hoc adjusted Tukey test, if data were normally distributed, or Kruskal–Wallis ANOVA with a post-hoc Dunn test, if data were not normally distributed, using OriginPro (Version 2022. OriginLab Corporation). The same statistical procedure was used to analyze the effects of PPP treatments on bacterial and fungal gene copy numbers on the cuticles. Permutation multivariate analysis of variance (NPMANOVA) based on Bray–Curtis similarity was performed using the software PAST 2.17c (Hammer et al., 2001) to analyze the differences between the different PPP treatments on the cuticular microbial communities and functions. The results were visualized by OriginPro (Version 2022. OriginLab Corporation). Noll et al. (2005) explained that relative abundances were calculated for each sample and visualized with OriginPro (Version 2022. OriginLab Corporation). Significantly distinctive cuticular bacterial and fungal genera of the PPP treatments were identified using indicator species analysis conducted using the “multipatt” function in the indicspecies package (de Cáceres and Legendre, 2009), which calculates indicator values with the “r.g.” function.

## 3. Results

### 3.1. PPP treatment significantly altered bacterial and fungal gene copy numbers

Our data showed that fungicides can significantly affect the bacterial and fungal gene copy numbers on the cuticle of honey bees. The fungicide Difcor® significantly reduced fungal gene copy numbers compared to control A (*p* = 0.04246), whereas neither the insecticide Steward® nor the mixture of Difcor® and Steward® affected fungal gene copy numbers (Supplementary Figure 1A).

**Figure 2:**
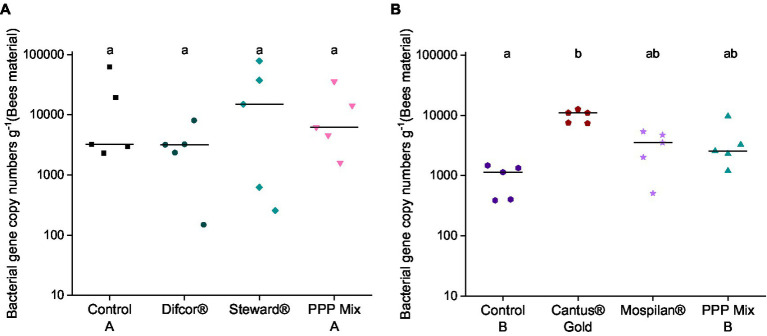
Bacterial gene copy **(A,B)** numbers after PPP treatment. PPP Difcor®, Steward®, or the combination of both (mix A) **(A)** and PPP Cantus® Gold, Mospilan® and the combination of both (mix B) **(B)** treatments (*n* = 5). Different letters indicate statistically significant differences according to the Dunn test (*p* = 0.05). If the letters are the same, treatments were not significant to each other, and if they were different, treatments were significantly different. The fungal gene copy numbers are shown in Supplementary Figure 1.

Bacterial gene copy numbers were not affected by the fungicide Difcor®, the insecticide Steward®, or PPP mix A treatments (Figure 2A). In contrast, the fungicide Cantus® Gold significantly increased the bacterial gene copy numbers compared to control B (*p* = 0.00167) (Figure 2B). The insecticide Mospilan® and the PPP mix B had no significant effect on bacterial gene copy numbers compared to the control B (Figure 2B). PPP mix B treatment increased fungal gene copy numbers in all treatment groups (Supplementary Figure 1B). The highest fungal gene copy numbers could be found in the fungicide Cantus® Gold treatment, which differed significantly from the control B (*p* = 0.01528) and the insecticide Mospilan®-treated group (*p* = 0.01815) (Supplementary Figure 1B).

### 3.2. PPP treatments showed different effects on bacterial and fungal community compositions

**Figure 3:**
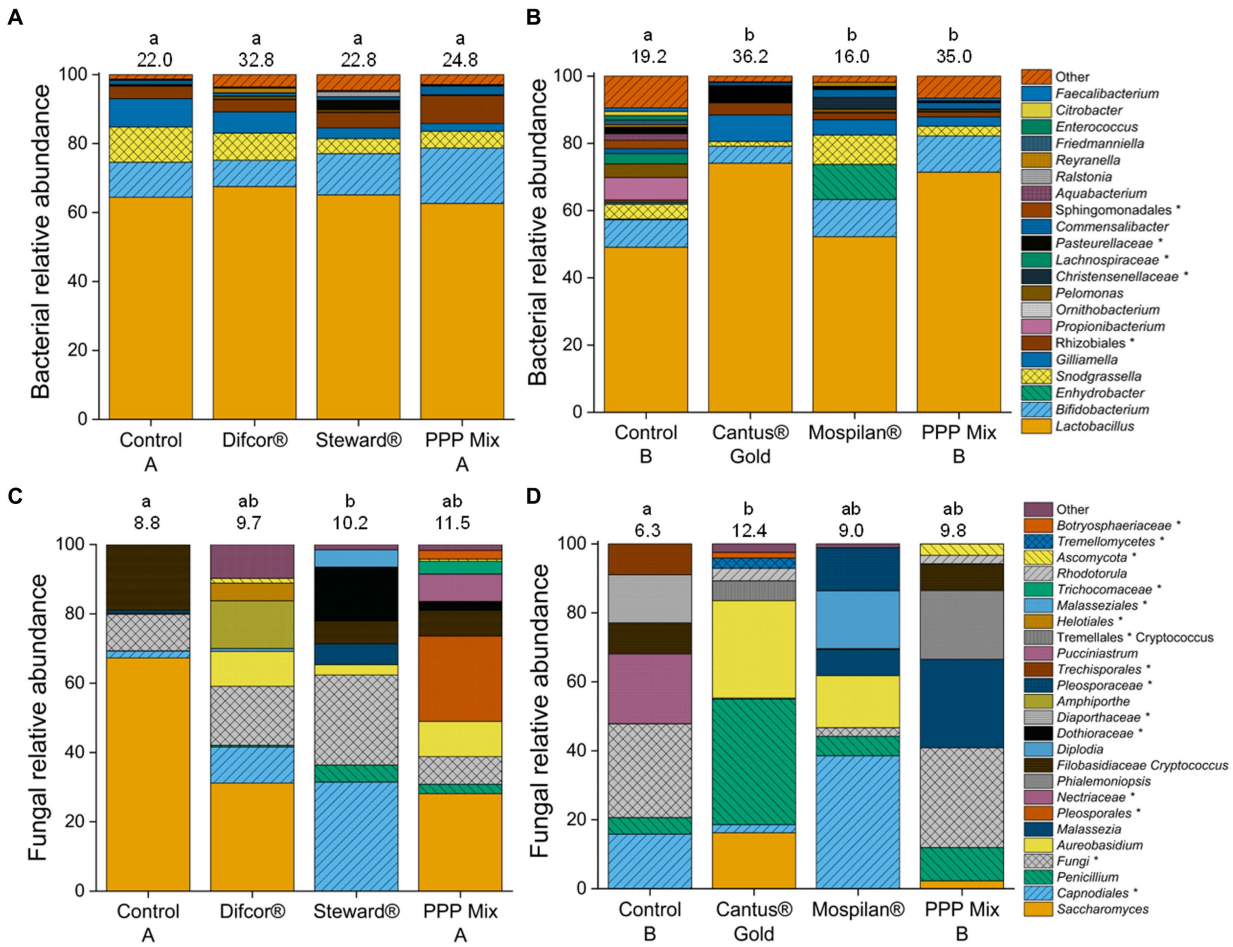
Relative sequence read abundance of the bacteria **(A,B)** and fungi **(C,D)** after PPP treatment. PPP Difcor®, Steward®, or the combination of both (mix A) **(A,C)** and PPP Cantus® Gold, Mospilan® and the combination of both (mix B) **(B,D)** treatments (*n* = 5). Bacterial species with a relative abundance of <1% were summarized as other. Complete bacterial abundance graphs without a summary of the low abundance can be found in Supplementary Figure 2. Fungal species with a relative abundance of <2% were summarized as other. Fully fungal abundance graphs without a summary of the low abundance can be found in different letters indicating statistically significant differences according to one-way non-parametric multivariate analysis (*p* = 0.05). If the letters are the same, treatments were not significantly different to each other, and if they were different, treatments were significantly different. The numbers above the bars reflect the respective OTU richness. Unclassified members of the taxon are marked with *.

The data indicate that the insecticide and fungicide treatments affect the fungal community composition. The fungal community composition was significantly altered by the insecticide Steward® treatment and differed significantly from control A (*p* = 0.0157). The fungicide Cantus® Gold significantly altered fungal community composition (*p* = 0.0239) in comparison with control B. Cantus® Gold, Mospilan®, and PPP mix B treatments significantly changed bacterial community composition in all treatments compared to control B (Figure 3; Supplementary Table 1).

In contrast, the insecticide Mospilan® and PPP mix B treatments did not lead to any alterations in the fungal community composition compared to the Cantus® Gold treatment and control B (Supplementary Table 1). The fungicide Difcor®, insecticide Steward®, and PPP mix A treatments did not significantly change the bacterial community composition (Figure 3; Supplementary Table 2). Bacterial and fungal OTU richness were not affected by the Difcor®, Steward®, and PPP mix A treatments [Dunn’s test (*p* = 0.05)] or Cantus® Gold, Mospilan®, and PPP mix B treatments [Tukey test (*p* = 0.05); Figure 3].

### 3.3. PPP treatments showed different effects on bacterial and fungal community functions

**Figure 4:**
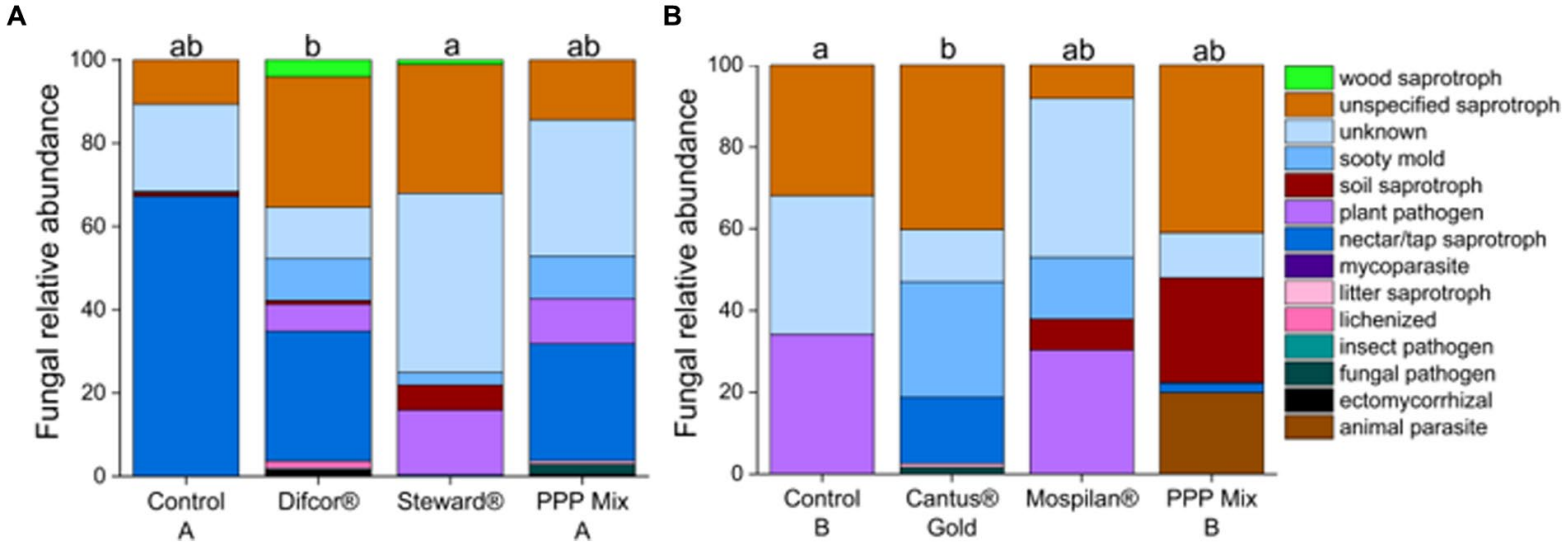
Fungal functional composition on genus level after PPP treatment. PPP Difcor®, Steward®, or the combination of both (mix A) **(A)** and PPP Cantus® Gold, Mospilan® and the combination of both (mix B) **(B)** treatments (*n* = 5). Different letters indicate statistically significant differences according to one-way non-parametric multivariate analysis (*p =* 0.05). If the letters are the same, treatments were not significant to each other, and if they were different, treatments were significantly different. Supplementary Tables 3, 4 provide which fungal genera were assigned to which functional traits.

According to our data, insecticides and fungicides had an impact on fungal and bacterial functional composition. The insecticide Steward® treatment significantly impacted the fungal functional composition compared to control A (*p* = 0.0154) (Figure 4A). Furthermore, the fungicide Cantus® Gold treatment significantly altered fungal functional community composition in comparison with control B (*p* = 0.0255) (Figure 4B).

Plant pathogenic fungi had increased sequence read abundance in the fungicide Difcor® (19%) and insecticide Steward® (22%)-treated bees in comparison with control A (<1%) (Figure 4A). Whereas sequence read abundance of plant pathogenic fungi was reduced in the treatment’s fungicide Cantus® Gold (2%), and PPP mix B (20%) or not altered in the insecticide Mospilan® (30%) treatment in comparison with control B (34%) (Figure 4B). The fungicide Difcor®-treated bees’ cuticular fungal microbiome was highly associated with the fungal genus *Amphiporthe* (Table 3), and the fungicide Cantus® Gold-treated bees’ cuticular fungal microbiome was associated with the genus *Saccharomyces.* The insecticides Steward® and PPP mix A, and control A, as well as the insecticides Mospilan®, PPP mix B, and control B, were not associated with any fungal indicator species. Fungicide Difcor® and PPP mix A did not differ in the fungal functional community composition compared to control A and the insecticide Steward® treatment (Figure 4A). The same could be observed for the insecticide Mospilan® and PPP mix B in comparison with control B and the Cantus® Gold treatment (Figure 4A).

**Table 3:** Indicator species analysis of cuticular bacterial and fungal community members after PPP treatment.

	PPP treatment	Stat	Value of *p*	Significance	Family	Genus	Function/bee location
Bacteria	Mix A	0.703	0.0059	**	*Acetobacteraceae*	*Commensalibacter*	Nitrate and nitrite respiration; Bee gut
	Cantus® Gold	0.480	0.0314	*	Lactobacillales*	Lactobacillales*	NA; Bee gut
		0.399	0.0046	**	*Flavobacteriaceae*	*Ornithobacterium*	Aerobic chemoheterotrophy
	Mix B	0.739	0.0054	**	*Orbaceae*	*Frischella*	NA; Bee gut
	Mospilan®	0.659	0.0077	**	*Neisseriaceae*	*Snodgrassella*	NA; Bee gut
Fungi	Difcor®	0.447	0.0088	**	*Gnomoniaceae*	*Amphiporthe*	Unspecified saprotroph
	Cantus® Gold	0.558	0.0352	*	*Saccharomycetaceae*	*Saccharomyces*	Nectar/tap saprotroph

The Stat value and significance of each species and its function are shown, ordered after the Stat-value. Unclassified members of the taxon are marked with *. Significant levels: ‘**’ 0.01 ‘*’ 0.05 ‘.’ 0.1. NA: No function was assigned.

In contrast to the fungal community composition, only the insecticide Mospilan® treatment led to significant differences in the bacterial functional composition compared to control B (*p* = 0.0165) (Supplementary Figure 4B). Indicator analysis of Difcor®, Steward®, and PPP mix A treatments showed that *Commensalibacter* was highly associated with the bacterial community of the PPP mix A treatment (Table 3). The fungicide Difcor® treatment, the insecticide Steward® treatment, and control A did not bear any bacterial indicators. The cuticular microbiome of Cantus® Gold-treated bees was significantly associated with unclassified members of the order *Lactobacillales*. Furthermore, a weaker association with *Ornithobacterium* could be observed (Table 3). The cuticular bacterial microbiome of Mospilan®-treated bees’ high associations with the genus *Snodgrassela* was observed. The cuticular bacterial microbiome of the combined treatment with PPP mix B was highly associated with the genus *Frischella* (Table 3). The fungicide Difcor®, insecticide Steward®, and PPP mix A treatments compared to control A (Supplementary Figure 4A), as well as the fungicide Cantus® Gold and PPP mix B treatments, did not affect the bacterial functional community composition compared to control B and the insecticide Mospilan® treatment (Supplementary Figure 4B).

## 4. Discussion

Tremendous effects on the fungal microbiome could be observed for all treatments. Moreover, single pesticide treatments such as the fungicide Difcor® significantly reduced the fungal gene copy numbers (Supplementary Figure 1B). The insecticide Steward® led to significant alterations in the fungal community composition and function (Figures 3B; Supplementary Figure 4A).

Fungicide Cantus® Gold, insecticide Mospilan®, and PPP mix B treatments increased bacterial and fungal gene copy numbers (Figures 2C,D; Supplementary Figure 1B), and all treatments significantly altered the bacterial community composition (Figure 3C; Supplementary Table 1). The insecticide Mospilan® led to significant changes in the bacterial functional composition (Supplementary Figure 4B). Fungicide Cantus® Gold had tremendous effects on the fungal cuticular community and functional composition (Figure 3D; Supplementary Figure 4B).

The fungicide Difcor® and insecticide Steward® treatments significantly impacted the cuticular bacterial community composition (Figure 3A; Supplementary Table 1A), while the bacterial gene copy numbers and bacterial community functions were unaffected (Figure 2A). The genus *Commensalibacter* was identified as an indicator taxa of the cuticular bacterial microbiome after PPP mix A treatment (Table 3). Members of the genus *Commensalibacter* were previously described as a core member of the honey bees’ gut microbiome (Martinson et al., 2011; Kwong and Moran, 2016) and as an essential part of the honey bees’ microbial ecosystem (Wu et al., 2022).

The fungicide Difcor®, insecticide Steward®, and PPP mix A treatments caused a reduction of the fungal gene copy numbers compared to the control A. Moreover, the fungicide Difcor®, insecticide Steward®, and PPP mix A treatments led to a higher diversity of the cuticular fungal community composition (Figure 3B). Similar results could be observed for the intestine microbiome of bees after treatment with pesticides (Syromyatnikov et al., 2020). Similarly, the fungicide Difcor® significantly reduced the fungal gene copy numbers (Supplementary Figure 1B), and members of the genus *Amphiporthe* were significantly associated with this treatment. However, a recent study found that members of the same family, *Valsaceae,* were described as sensitive toward difenoconazole (Silva-Campos et al., 2022), indicating an ambivalent biocide response in this family. Furthermore, Steward® significantly changed cuticular fungal community composition and function (Figures 3B, 4A; Supplementary Table 2). *Saccharomyces* was not found in the insecticide Steward®-treated groups, indicating that members of *Saccharomyces* were highly sensitive to the insecticide Steward®. However, *Saccharomyces* was the most abundant genus in control A (Figure 3). Unclassified members of the genera *Dothioraceae* and *Capnodiales* were the most abundant species in the Steward®-treated cuticular fungal microbiomes (Figure 3B). Members of the genus *Dothioraceae* were already found in the guts of nectar-collecting *Apis cerana* (Basukriadi et al., 2010). *Capnodiales* was previously described as increasing abundance of chlorothalonil-based fungicides in field-relevant level-treated hives (Steffan et al., 2017). Even though the active ingredient differs from the insecticide Steward®, this result indicates that *Capnodiales* benefits from the treatment with PPP either directly by the inactivation of competitors or their predators and/or indirectly by microbial metabolites or degradation products released from PPP-sensitive species. Nectar/tap saprotrophs were two-thirds the most abundant group in control A. Nectar/tap saprotrophic fungi were reduced in the fungicide Difcor®, insecticide Steward®, and PPP mix A treatments compared to control A (Figure 4A). Those were reduced by less than 1% in the insecticide Steward® treatment, indicating that this insecticide alone already caused this reduction of nectar/tap saprotrophic fungi. The active ingredient of Steward® is indoxacarb; the insecticidal activity occurs by blocking the sodium channels within the nervous system of insects (Wing et al., 2000). Even though mycorrhizal fungi are described as playing an important role in balancing salinity within the environment and the use of sodium for signaling, no voltage-gated sodium channels have been found for fungi (Scharnagl et al., 2017). Thereby, we are the first to describe this non-target effect of indoxacarb on nectar/tap saprotrophic fungi. To date, honey analyses have not shown any negative effects of the use of any of these fungicides. However, based on our study, existing data and set-ups should be revisited in detail if they do affect honey quality.

Interestingly, plant pathogens gained abundance in the cuticular fungal community of the fungicide Difcor® and insecticide Steward®-treated bees in comparison with control A (Figure 4A). Moreover, sooty mold fungi were increased in the cuticular fungal community of fungicide Difcor®, insecticide Steward®, and PPP mix A-treated bees. Those fungi are reported to show resistance against difenoconazole (Difcor®) (Yang et al., 2019). Moreover, those were described as showing cross-resistance even for fungicides with a different mode of action (Yang et al., 2019), which might be the reason for their high sequence read abundances in the insecticide Steward®-treated bees (Figure 4A). Similar to the cuticular fungal community composition, the diversity of functional composition was increased due to the fungicide Difcor®, insecticide Steward®, and PPP mix A treatments, which were already observed for the intestinal microbiome of bees (Silva-Campos et al., 2022).

The fungicide Cantus® Gold, insecticide Mospilan®, and PPP mix B treatments shifted the bacterial and fungal cuticular community composition. Fungicide Cantus® Gold significantly increased bacterial and fungal gene copy numbers (Figures 2C,D). Fungicide Cantus® Gold, insecticide Mospilan®, and PPP mix B treatments significantly changed cuticular bacterial community composition (Figure 3C; Supplementary Tables 1, 2). Although the composition of the bacterial community was altered by the treatments with the fungicide Cantus® Gold, the insecticide Mospilan®, and the PPP mixture B, only the insecticide Mospilan® showed a significant change in the functional composition of the cuticular bacteria (Supplementary Figure 4B). Alberoni et al. (2021) observed a significant decrease in the neonicotinoid-treated groups and a compirsed functionality of the gut microbiome of bees. This is in line with our results for the bacterial functional community composition after treatment with the insecticide Mospilan.

The indicator analysis for fungicide Cantus® Gold, insecticide Mospilan®, and PPP mix B-treated bees revealed indicator species for all treatments. For example, *Snodgrassella* was significantly associated with the insecticide Mospilan®-treated bees (Table 3), while *Frischella* was significantly associated with the PPP mix B. Both genera are dominant intestinal bacteria of bees (Babendreier et al., 2007). *Ornithobacterium* and unclassified members of the order *Lactobacillales* were significantly associated with the fungicide Cantus® Gold treatment. *Lactobacillales* are also known members of the bee’s gut microbiome (Babendreier et al., 2007; Martinson et al., 2011; Kwong and Moran, 2013). As gut microbiota are specialized to an ecological niche, it is likely to find those in another hive niche of its host species (Anderson et al., 2011). Furthermore, it was shown that grooming plays a role in implementing the bees’ gut microbiome (Powell et al., 2014). Thereby, the grooming processes of bees could also lead to the distribution of gut-associated bacteria on the cuticular.

The fungicide Cantus® Gold treatment significantly altered the cuticular fungal community composition of the bees compared to control B (Figure 3D; Supplementary Table 1). The insecticide Mospilan® and PPP mix B did not significantly differ from control B or the fungicide Cantus® Gold treatment. The fungal functional cuticular community composition of the fungicide Cantus® Gold treatment differed significantly from control B (Figure 4B). The relative sequence read abundance of sooty mold and nectar/tap saprotrophic fungi was significantly increased compared to the insecticide Mospilan® and PPP mix B treatments. Moreover, plant pathogenic fungi were significantly reduced by the fungicide Cantus® Gold from one-third to less than 1% (Figure 4B). It is known that fungicide treatment alters the hive fungal community composition by introducing residues from pollen or bees (Sammataro et al., 2012). Yoder et al. (2013) described that the mixture of boscalid and pyraclostrobin did alter the fungal community of bee bread. Pyraclostrobin is a strobilurin and belongs to the same chemical group as dimoxystrobin, which forms together with boscalid, the active ingredient of the fungicide Cantus® Gold. We already described that the in-hive microbiome is closely connected to the bees’ microbiome. Therefore, it is likely that this is also the case for fungi. *Saccharomyces* was identified as an indicator of the fungicide Cantus® Gold-treated cuticular fungal microbiomes (Table 3) and is described as nectar/tap saprotrophic. Hnátová et al. (2003) described that mutants of *Saccharomyces* show resistance against strobilurin fungicides. In our experiments, we did not analyze the effects of the fungicides, the insecticides, or their combinations on honey bee health or honey production. Based on studies by Degrandi-Hoffman et al. (2015), it can be assumed that the fungicide boscalid, which was also applied in our study, can increase pathogens such as deformed wing virus or black queen cell virus. Furthermore, Pettis et al. (2013) demonstrated the effect of boscalid ingestions on the probability of Nosema infections. Similar to fungicides, neonicotinoids can affect honey bee health. Harwood and Dolezal (2020) showed negative effects on hemocyte differentiation and function following neonicotinoid application. Brandt et al. (2016) demonstrated that neonicotinoids can reduce hemocyte density, encapsulation response, and antimicrobial activity in honey bee. In order to investigate the further effects of PPPs on bee health, experiments using a different experimental design compared to our study should be performed.

## 5. Conclusion

Our results have demonstrated for the first time that both insecticides and fungicides can have adverse effects on the microbiome on the cuticle of honey bees, which has to date been completely neglected when investigating the side effects of PPPs. The cuticle microbiome may serve important functions as a barrier against harmful microbes. A change in the composition of the microbiome may have severe effects on honey bee health, which might only become apparent long after the collection or consumption of the respective insecticides or fungicides. The insecticide Steward® with the active substance indoxacarb and the fungicide Cantus® Gold with the active substances boscalid and dimoxystrobin are the most frequent residues in beebread, altering the fungal community composition of honey bee cuticles significantly. The neonicotinoid Mospilan® with the active substance acetamiprid significantly affected bacterial functional community composition. Mixtures of fungicides and insecticides could enhance the side effects of single substances, which have rarely been observed because fungicides are generally believed to be harmless to bees and other pollinators. In particular, fungal cuticular community composition was affected, showing a phylogenetic diversification due to the PPP mix treatments and an increase in pathogenic fungi on the bees’ cuticle. Our results urge more studies on side effects on honey bees and other bees caused by the interaction of insecticides and fungicides and demonstrate that the microbiome of the cuticle is a promising site for investigation because it is susceptible to the actions of PPPs.

## Data availability statement

The datasets presented in this study can be found in online repositories. The microbiome’s bacterial 16S rRNA and fungal ITS gene sequences were deposited in the NCBI nucleotide sequence databases (https://www.ncbi.nlm.nih.gov/) under accession PRJNA880009.

## Ethics statement

Ethical approval was not required for the study involving animals in accordance with the local legislation and institutional requirements because our protocols comply with standard welfare practice in our field. The experiment involved honey bee from an apiary dedicated to research.

## Author contributions

FR: Funding acquisition, Investigation, Supervision, Validation, Writing – original draft, Data curation, Formal analysis, Methodology, Visualization. AS: Data curation, Formal analysis, Investigation, Methodology, Writing – review & editing. LS: Data curation, Formal analysis, Investigation, Methodology, Writing – review & editing, Software, Validation, Visualization. MT: Writing – review & editing, Conceptualization. RS: Conceptualization, Writing – review & editing, Funding acquisition, Project administration, Resources, Supervision. MN: Conceptualization, Funding acquisition, Project administration, Resources, Supervision, Investigation, Validation, Writing – original draft.
