# Supplementary material for: Fungicides and insecticides can alter the microbial community on the cuticle of honey bees
Source: Front Microbiol. 2023 Oct 30;14:1271498. doi: 10.3389/fmicb.2023.1271498 (PMC10642971; doi:10.3389/fmicb.2023.1271498)
Supplement: Supplementary file 1 [file Data_Sheet_1.pdf]

Supplementary Material

1 Supplementary Tables

Supplementary Table 1. One-way non-parametric multivariate analysis of Cantus® Gold, Mospilan®, and PPP mix B treatments. One-way non-parametric multivariate analysis based on the Bray-Curtis similarities of the bacterial and fungal community compositions after the treatment with PPP Cantus® Gold, Mospilan® and the combination of both (Mix B) treatment (n = 5).

		Control B	Cantus® Gold	Mospilan®	PPP Mix B
Bacterial community composition	Control B	0	0.0164*	0.025*	0.0093**
	Cantus® Gold	0.0164*	0	0.4771	0.3976
	Mospilan®	0.025*	0.4771	0	0.5419
	PPP Mix B	0.0093**	0.3976	0.5419	0
		Control Mix B	Cantus® Gold	Mospilan®	PPP Mix B
Fungal community composition	Control B	0	0.0239*	0.5655	0.1474
	Cantus® Gold	0.0239*	0	0.2633	0.0623
	Mospilan®	0.5655	0.2633	0	0.3268
	PPP Mix B	0.1474	0.0623	0.3268	0

Significant levels: '***' 0.001 '**' 0.01 '*' 0.05

Supplementary Table 2. One-way non-parametric multivariate analysis of the Difcor®, Steward®, and PPP mix A treatments. One-way non-parametric multivariate analysis based on the Bray-Curtis similarities of the bacterial and fungal community compositions after the treatment with PPP Difcor®, Steward® or the combination of both (Mix A) (n = 5).

		Control A	Difcor®	Steward®	PPP Mix A
**Bacterial community composition**	**Control A**	0	0.0208	0.7677	0.2952
	**Difcor®**	0.0208	0	0.6042	0.2888
	**Steward®**	0.7677	0.6042	0	0.6577
	**PPP Mix A**	0.2952	0.2888	0.6577	0
		**Control A**	**Difcor®**	**Steward®**	**PPP Mix A**
**Fungal community composition**	**Control A**	0	0.2123	0.0157 *	0.2757
	**Difcor®**	0.2123	0	0.3537	0.6881
	**Steward®**	0.0157 *	0.3537	0	0.2236
	**PPP Mix A**	0.2757	0.6881	0.2236	0

Significant levels: 0 ‘***’ 0.001 ‘**’ 0.01 ‘*’ 0.05 ‘.’ 0.1 ‘’ 1

Supplementary Table 3. Overview of the relative abundance of the genera and their respective primary lifestyle for the treatment groups Difcor®; Steward®, PPP Mix B and the respective control.

Genus	Primary lifestyle	Control A	Difcor®	Steward®	PPP Mix A
Alternaria	plant pathogen	0	0	0	0
Amphiporthe	unspecified saprotroph	18.9404	0	6.6248	7.5004
Amylostereum	wood saprotroph	0	5.0963	0	0
Ascochyta	plant pathogen	0.1040	0.4912	4.8929	2.6975
Ascomycota *	unknown	0	0.4268	0	0
Aureobasidium	sooty mold	0	0.0164	0	0
Basidiomycota *	unknown	0	0.2716	0	0
Bettsia	animal parasite	0.0002	0	0	0
Bionectriaceae *	insect pathogen	0	0	0	0
Botryosphaeriaceae *	fungal pathogen	0	0	1.1276	0
Botrytis	plant pathogen	0	0.0017	0	0
Candida	nectar/tap saprotroph	0	0	0.0002	3.6945
Capnodiales *	unknown	0	0.7184	0	0
Ceratobasidiaceae *	ectomycorrhizal	0	1.3950	0	0
Chaetomiaceae *	unspecified saprotroph	0	0	0.0013	24.6038
Cladonia	lichenized	0	0	4.9842	0

Cladosporium	litter saprotroph	0	0	0	7.8985
Cortinarius	ectomycorrhizal	0	2.2129	0	0
Cystofilobasidium	litter saprotroph	1.9742	10.3854	31.4769	0.0007
Dasyscyphella	wood saprotroph	0	0	0.0011	0
Davidiellaceae *	litter saprotroph	67.2751	31.1943	0.0004	28.1207
Diaporthaceae *	plant pathogen	0	0.6258	0	0
Diplodia	plant pathogen	0.0055	0	0	0
Dothideales *	plant pathogen	0	0.0009	0	0
Dothioraceae *	plant pathogen	0	13.7599	0	0
Endoconidioma	wood saprotroph	0.8453	0	5.9956	0
Filobasidiaceae *Cryptococcus*	unknown	0.2987	10.0461	2.9657	10.1624
Fungi *	unspecified saprotroph	0	0	0	0
Fusarium	plant pathogen	0.0043	0	0	0
Geastrum	litter saprotroph	0	0	0	0.2132
Helotiales *	plant pathogen	0	0	0	0.4850
Leotiomycetes *	unknown	0.0066	0	0	0
Leucosporidiaceae *	unknown	0	0	0	0
Leucosporidium	soil saprotroph	0	0	0	0
Malassezia	soil saprotroph	0.0008	0.0020	0	0
Malasseziales *	unknown	0	0	0	0.4391

Metschnikowia	nectar/tap saprotroph	0	0.0026	0	0
Microbotryomycetes *	mycoparasite	0.0022	0	15.5403	2.5000
Montagnulaceae *	unknown	0	0	0	0
Mortierella	soil saprotroph	0.0027	0	0.3964	0
Nectriaceae *	plant pathogen	0	0	0	0
Oidiodendron	soil saprotroph	0	0	0	0
Penicillium	unspecified saprotroph	0	0	0	0
Phialemoniopsis	animal parasite	0	1.8385	0	0
Phlebia	wood saprotroph	10.5340	17.0442	25.9924	7.9831
Pleosporaceae *	plant pathogen	0.0004	0	0	0
Pleosporales *	unknown	0	0	0	0
Pseudotaeniolina	litter saprotroph	0.0012	0	0	0
Pucciniastrum	plant pathogen	0	1.1391	0	0
Rhodotorula	unspecified saprotroph	0	0.8531	0	0
Saccharomyces	nectar/tap saprotroph	0	0	0.0001	0
Thanatephorus	plant pathogen	0	0	0	0.5570
Tranzschelia	plant pathogen	0	0.5139	0	0
Trechisporales *	unknown	0	0	0	0
Tremellales * *Cryptococcus*	unknown	0	0.0013	0	0

Tremellomycetes *	unknown	0	0	0	2.5000
Trichocomaceae *	unspecified saprotroph	0.0045	1.3644	0	0.6441
Trichoderma	mycoparasite	0	0.5985	0	0

Unclassified members of the taxon are marked with *.

Supplementary Table 4. Overview of the relative abundance of the genera and their respective primary lifestyle for the treatment groups Cantus® Gold; Mospilan®, PPP Mix B and the respective control.

**Genus**	**Primary lifestyle**	**Control B**	**Cantus® Gold**	**Mospilan®**	**PPP Mix B**
Alternaria	plant pathogen	0	0	0.0001	0.0091
Amphiporthe	unspecified saprotroph	0	0	0	0
Amylostereum	wood saprotroph	0	0	0	0
Ascochyta	plant pathogen	0	0	1.1245	0
Ascomycota *	unknown	0	0.0014	0.0019	3.2946
Aureobasidium	sooty mold	0.0517	28.2572	15.0871	0.0004
Basidiomycota *	unknown	0	0	0	0
Bettsia	animal parasite	0	0.0065	0	0
Bionectriaceae *	insect pathogen	0	0	0	0
Botryosphaeriaceae *	fungal pathogen	0	1.6313	0	0
Botrytis	plant pathogen	0	0.0062	0	0
Candida	nectar/tap saprotroph	0	0	0	0

Capnodiales *	unknown	15.8484	2.3773	38.5780	0.0111
Ceratobasidiaceae *	ectomycorrhizal	0	0	0	0
Chaetomiaceae *	unspecified saprotroph	0	0	0	0
Cladonia	lichenized	0	0	0	0
Cladosporium	litter saprotroph	0	0.0007	0	0
Cortinarius	ectomycorrhizal	0	0	0	0
Cystofilobasidium	litter saprotroph	0	0.9299	0	0
Dasyscyphella	wood saprotroph	0	0	0	0
Davidiellaceae *	litter saprotroph	0.0003	0.0003	0	0
Diaporthaceae *	plant pathogen	13.9254	0	0	0.0004
Diplodia	plant pathogen	0	0	16.7570	0.0004
Dothideales *	plant pathogen	0	0.0009	0	0
Dothioraceae *	plant pathogen	0	0.0010	0	0
Endoconidioma	wood saprotroph	0	0	0	0
Filobasidiaceae *Cryptococcus*	unknown	9.0397	0.0011	0.0009	7.6830
Fungi *	unspecified saprotroph	27.1975	0.2649	2.5018	28.9372
Fusarium	plant pathogen	0	0	0	0
Geastrum	litter saprotroph	0	0	0	0
Helotiales *	plant pathogen	0	0	0	0.1041

Leotiomycetes *	unknown	0	0	0	0
Leucosporidiaceae *	unknown	0	1.4979	0	0
Leucosporidium	soil saprotroph	0	0.0008	0	0
Malassezia	soil saprotroph	0	0.0001	7.5494	25.6514
Malasseziales *	unknown	0	0	0	0
Metschnikowia	nectar/tap saprotroph	0	0.0062	0	0
Microbotryomycetes *	mycoparasite	0	0.0006	0	0
Montagnulaceae *	unknown	0	0	0	0
Mortierella	soil saprotroph	0	0	0	0
Nectriaceae *	plant pathogen	20.2132	0	0	0
Oidiodendron	soil saprotroph	0	0	0	0
Penicillium	unspecified saprotroph	4.7781	36.4027	5.6496	9.6326
Phialemoniopsis	animal parasite	0	0	0.0007	19.9144
Phlebia	wood saprotroph	0	0	0	0
Pleosporaceae *	plant pathogen	0	0	12.4502	0
Pleosporales *	unknown	0	0.0036	0.2984	0
Pseudotaeniolina	litter saprotroph	0	0	0	0
Pucciniastrum	plant pathogen	0	0	0	0
Rhodotorula	unspecified saprotroph	0.0005	3.5773	0.0001	2.4612

Saccharomyces	nectar/tap saprotroph	0.0093	16.2383	0.0004	2.2999
Thanatephorus	plant pathogen	0	0	0	0
Tranzschelia	plant pathogen	0	0	0	0
Trechisporales *	unknown	8.9360	0	0	0
Tremellales * *Cryptococcus*	unknown	0	5.7554	0	0
Tremellomycetes *	unknown	0	3.0380	0	0
Trichocomaceae *	unspecified saprotroph	0	0.0004	0	0
Trichoderma	mycoparasite	0	0	0	0

Unclassified members of the taxon are marked with *.

2 Supplementary Figures

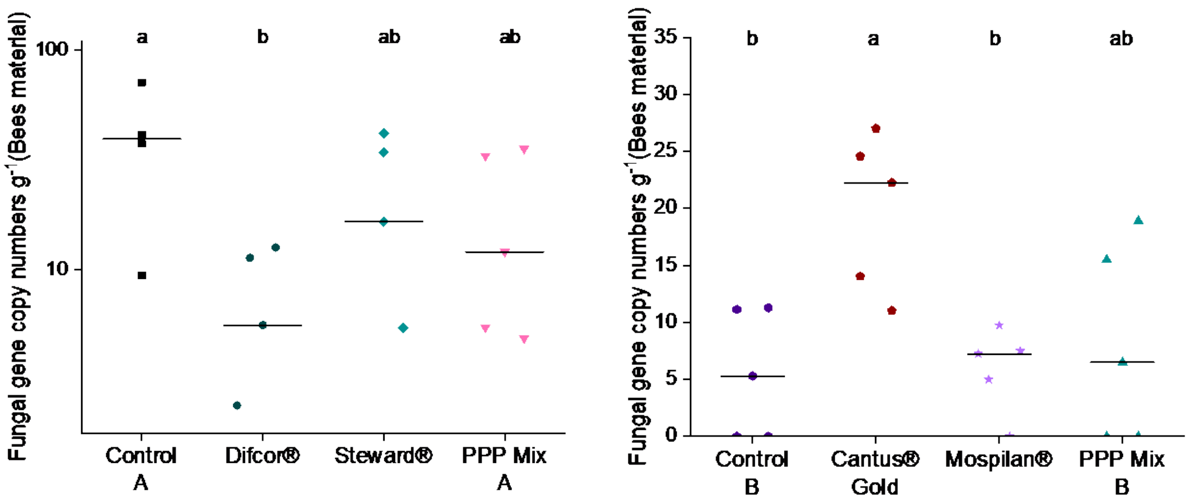

Supplementary Figure 1. Fungal gene copy (a; b) numbers after PPP treatment. PPP Difcor®, Steward® or the combination of both (Mix A) (a) and PPP Cantus® Gold, Mospilan® and the combination of both (Mix B) (b) treatment (n=5). One sample from control a was identified as significant outlier by Grubbs-Test ($p = 1.56 \times 10^{-8}$) and therefore excluded from the statistical tests and the plot. Both Cantus® Gold and Mospilan® treatment significantly influenced the fungal gene copy numbers (Kruskal-Wallis-ANOVA, $p = 0.05$).

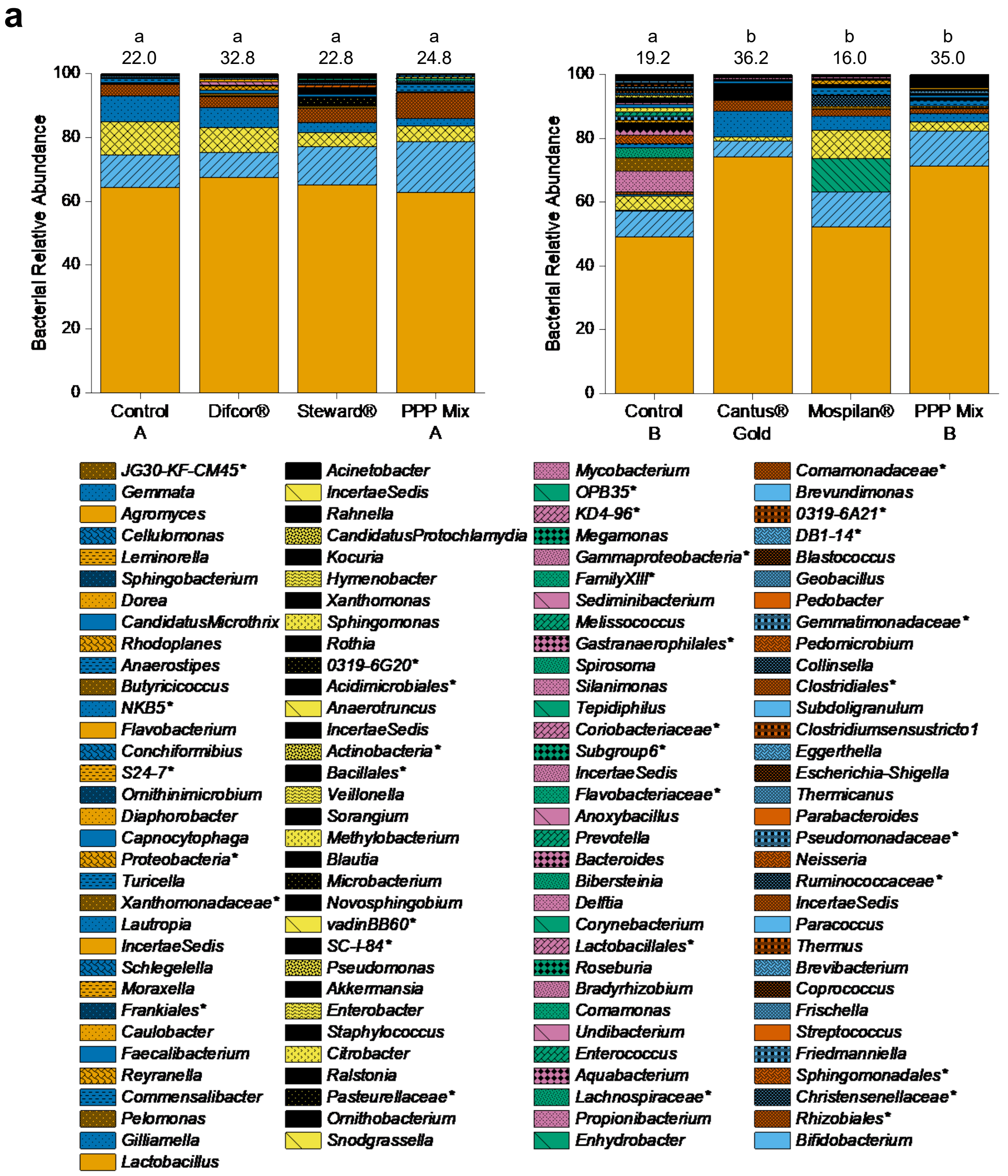

Supplementary Figure 2. Relative sequence read abundance of the bacteria (a; b) after PPP treatment. PPP Difcor®, Steward® or the combination of both (Mix A) (a) and PPP Cantus® Gold, Mospilan® and the combination of both (Mix B) (b) treatment (n=5). Different letters indicate statistically significant differences according to one-way non-parametric multivariate analysis (p = 0.05). The numbers above the bars reflect the respective OTU Richness. Unclassified members of the taxon are marked with *.

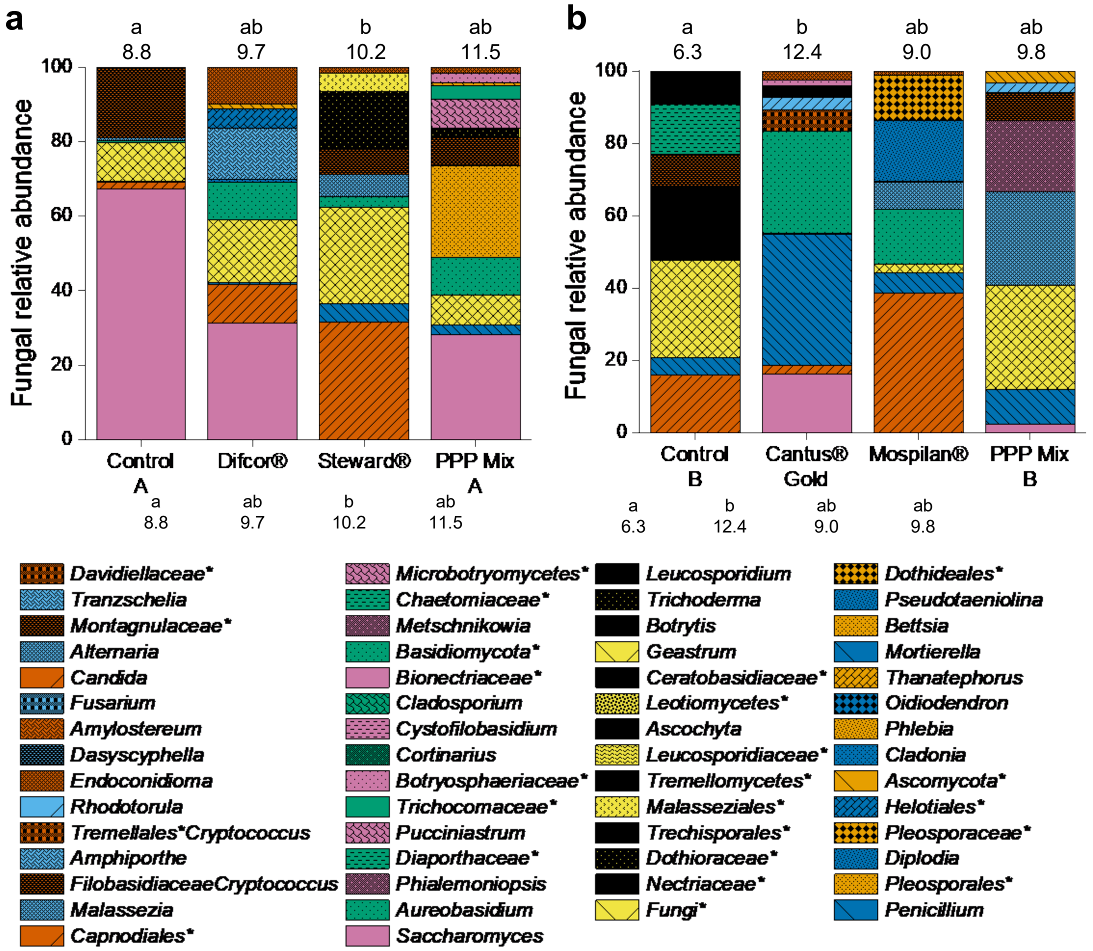

Supplementary Figure 3. Relative sequence read abundance of the fungi (a; b) after PPP treatment. PPP Difcor®, Steward® or the combination of both (Mix A) (a) and PPP Cantus® Gold, Mospilan® and the combination of both (Mix B) (b) treatment (n=5). Different letters indicate statistically significant differences according to one-way non-parametric multivariate analysis (p = 0.05). The numbers above the bars reflect the respective OTU Richness. Unclassified members of the taxon are marked with *.

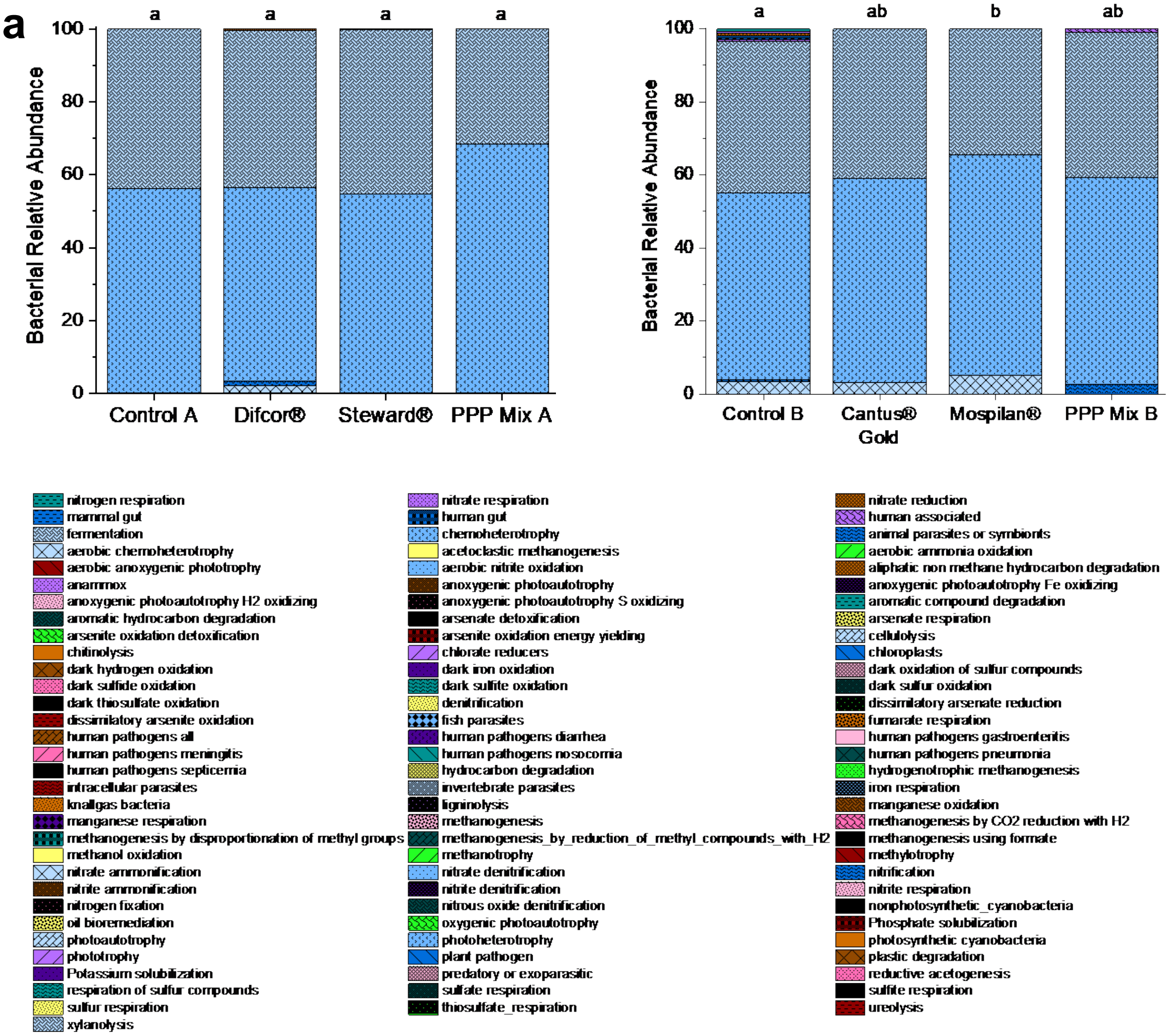

Supplementary Figure 4. Bacterial functional composition on OTU level after PPP treatment. PPP Difcor®, Steward® or the combination of both (Mix A) (a) and PPP Cantus® Gold, Mospilan® and the combination of both (Mix B) (b) treatment (n=5). Different letters indicate statistically significant differences according to one-way non-parametric multivariate analysis (*p* = 0.05).
